# Amiodarone Inhibits Apamin-Sensitive Potassium Currents

**DOI:** 10.1371/journal.pone.0070450

**Published:** 2013-07-29

**Authors:** Isik Turker, Chih-Chieh Yu, Po-Cheng Chang, Zhenhui Chen, Yoshiro Sohma, Shien-Fong Lin, Peng-Sheng Chen, Tomohiko Ai

**Affiliations:** 1 Krannert Institute of Cardiology and Division of Cardiology, Department of Medicine, Indiana University School of Medicine, Indianapolis, Indiana; 2 Division of Cardiology, Department of Medicine, National Taiwan University, Taipei, Taiwan; 3 Department of Pharmacology, Keio University School of Medicine, Shinjuku, Tokyo, Japan; Georgia State University, United States of America

## Abstract

**Background:**

Apamin sensitive potassium current (*I*_KAS_), carried by the type 2 small conductance Ca^2+^-activated potassium (SK2) channels, plays an important role in post-shock action potential duration (APD) shortening and recurrent spontaneous ventricular fibrillation (VF) in failing ventricles.

**Objective:**

To test the hypothesis that amiodarone inhibits *I*_KAS_ in human embryonic kidney 293 (HEK-293) cells.

**Methods:**

We used the patch-clamp technique to study *I*_KAS_ in HEK-293 cells transiently expressing human SK2 before and after amiodarone administration.

**Results:**

Amiodarone inhibited *I_KAS_* in a dose-dependent manner (IC_50_, 2.67±0.25 µM with 1 µM intrapipette Ca^2+^). Maximal inhibition was observed with 50 µM amiodarone which inhibited 85.6±3.1% of *I_KAS_* induced with 1 µM intrapipette Ca^2+^ (n = 3). *I_KAS_* inhibition by amiodarone was not voltage-dependent, but was Ca^2+^-dependent: 30 µM amiodarone inhibited 81.5±1.9% of *I*_KAS_ induced with 1 µM Ca^2+^ (n = 4), and 16.4±4.9% with 250 nM Ca^2+^ (n = 5). Desethylamiodarone, a major metabolite of amiodarone, also exerts voltage-independent but Ca^2+^ dependent inhibition of *I*_KAS_.

**Conclusion:**

Both amiodarone and desethylamiodarone inhibit *I*_KAS_ at therapeutic concentrations. The inhibition is independent of time and voltage, but is dependent on the intracellular Ca^2+^ concentration. SK2 current inhibition may in part underlie amiodarone's effects in preventing electrical storm in failing ventricles.

## Introduction

Heart failure is a major public health problem with 300,000 directly attributable deaths annually, in the United States alone. It has a prevalence of 5.8 million in the U.S. and over 23 million worldwide [1]. Ventricular arrhythmias are a major cause of morbidity and mortality in heart failure [2]. Today, many patients with heart failure receive an implantable cardioverter defibrillator (ICD) for primary or secondary prevention of arrhythmic death. However, ICD itself does not reduce the incidence of arrhythmias. Electrical storm (ES) defined as recurrent ventricular arrhythmias in a short period of time, remains a frequent complication and a strong independent predictor of poor outcome even in patients with ICDs [3], [4]. Amiodarone is effective in the treatment of recurrent ventricular tachycardia or fibrillation [5] and is commonly used as the first line therapy for ES [6], [7]. However, the mechanism behind amiodarone's effectiveness in treating ES remains poorly understood.

Ca^2+^ activated K^+^ channels integrate intracellular calcium handling with membrane repolarization in various tissues including brain, peripheral nerve, endothelium, leukocytes, erythrocytes, heart, skeletal and smooth muscle [8]. They are classified into three types based on their conductance pattern: large (BK), intermediate (IK) and small (SK) conductance Ca^2+^ activated K^+^ channels. SK channels show weak voltage dependence, susceptibility to the bee venom toxin apamin, and they are highly Ca^2+^ sensitive [9]. However, the role of these channels in the heart is poorly understood.

Xu *et al.* identified three isoforms of SK channels (SK1, SK2 and SK3) in the mouse and human heart, and found that they play important roles in the maintenance of action potential duration (APD) in atrial myocytes and pacemaking tissues [10]. Subsequently, the same group demonstrated that mice engineered to lack SK2 have prolonged atrial APD and higher susceptibility to atrial fibrillation [11]. Interestingly, SK2 expression is strikingly higher in normal mouse, cat and human atria than their respective ventricles [10]. This preferential expression led researchers to propose SK2 as a target for treating atrial arrhythmias without ventricular proarrhythmic risk. However, in our recent study, we demonstrated that SK2 expression is significantly upregulated in failing ventricles compared to normal ones [12]. Moreover, upregulation of SK2 channels contributes to the development of ES in heart failure [13], [14] and apamin, a specific inhibitor of SK2 channels [15], [16], can prevent the post-shock APD shortening and ES in failing rabbit ventricles [13].

These findings led us to hypothesize that amiodarone, the most effective and commonly used antiarrhythmic agent for the treatment of ES, can inhibit SK2 channels. However, amiodarone also inhibits various ion currents (e.g., *I*_Na_, *I*_Ca,L_, *I*_Ks_, *I*_Kr_, and *I*_to_) as well as β-adrenergic receptors. One possible approach to test the effects of amiodarone on SK2 currents is to first inhibit other major repolarization currents (such as *I*_Ks_, and *I*_Kr_) before administering amiodarone in cardiac cells. However, we found that chromanol 293B and E-4031 (known inhibitors of *I*_Ks_ and *I*_Kr_) are also inhibitors of SK2 channels. Using these two drugs in cardiac cells may prevent us to accurately study the effects of amiodarone on SK2 currents. Therefore, to test amiodarone's effects by itself on SK2 channels, we expressed the human SK2 in human embryonic kidney 293 (HEK-293) cells by transiently transfecting its coding gene *KCNN2*, variant 1. We then used the patch clamp method to demonstrate that both amiodarone and desethylamiodarone, a major metabolite of amiodarone, inhibit apamin-sensitive SK2 currents (*I*_KAS_) at therapeutic concentrations (10–20 µM). In addition, we confirmed that amiodarone prevents postshock APD shortening in failing rabbit ventricles. Our findings suggest that the antiarrhythmic action of amiodarone in the treatment of ES is, in part, due to *I*_KAS_ inhibition.

## Materials and Methods

All experimental protocols were approved by the Institutional Committee of Animal Use and Care, and the Institutional Committee of Human and DNA Research at Indiana University.

### HEK-293 cell preparation and transfection

HEK-293 cells obtained from American Type Cell Culture were grown at 37°C and 5% CO_2_ in Dulbecco's Modified Eagle's Medium supplemented with 10% FBS. *KCNN2*, the gene encoding human small conductance Ca^2+^-activated K^+^ channel, subfamily N, member 2 (SK2), transcript variant 1 (Gene Bank Accession # NM_021614.2), was obtained from OriGene (Rockville, MD) and constructed in pCMV6-XL plasmids. Naïve HEK-293 cells in 35 mm dishes were co-transfected with 4 µg of the expression construct and 0.5 µg of pEGFP-C3 plasmid using Lipofectamine 2000 (Invitrogen, Carlsbad, CA). Transfected cells were incubated at 37°C and 5% CO_2_ for 48 hours prior to patch-clamp experiments. Cells showing green fluorescence were chosen to study the effects of amiodarone on SK2.

### Expression of SK2 and *I_KAS_*

The HEK-293 cells transfected only with pEGFP-C3 were used as negative control for the patch clamp experiments. These did not generate significant *I*_K_ with 1 µM intrapipette Ca^2+^ (**Figure S1 in Data S1**). Since apamin effectively suppressed almost all K^+^ currents in the transfected cells, nearly all currents in this study were apamin sensitive (*I*_KAS_). Apamin concentration used in our study (100 nM) was higher than the concentration shown to exert maximal inhibition on SK2 channels in other studies [15], [17]. DMSO as a vehicle at the concentration of 0.1% had no effects on *I*_KAS_ (data not shown).

### Patch-clamp Experiments

Effects of amiodarone on *I*_KAS_ were studied using the whole-cell patch-clamp technique. Pipette resistances were 2–4 MΩ when filled with pipette solution. After whole-cell patch was obtained in Tyrode's solution, chamber bath was changed to a solution containing N-methylglucamine. Capacitance currents were monitored with repetitive 5 mV pulses for at least five minutes to measure the cell capacitance. Whole-cell compensation was not used for *I*_KAS_ measurements. All experiments were performed at 36°C. Voltage pulse protocols were generated with an Axopatch 200B amplifier using pCLAMP-10 software (Molecular Devices/Axon, Sunnyvale, CA). The data were filtered with a built-in four-pole low-pass Bessel filter at 2 kHz and digitized at 5 kHz. Extracellular solution contained (in mM): NMG, 140; KCl, 4; MgCl_2_, 1; glucose, 5; and HEPES, 10 (pH 7.4 with HCl). Pipette solution contained (in mM): potassium gluconate, 144; MgCl_2_, 1.15; EGTA, 1; HEPES, 10; MgATP, 2; and CaCl_2_; 0.85 (pH 7.25 with KOH). This composition yields 1 µM free Ca^2+^ at 36°C based on the calculation method by Bers *et al*. [18]. For study of Ca^2+^-dependency, various combinations of EGTA and CaCl_2_ were used in the pipette solution to achieve different intracellular [Ca^2+^].

Since SK2 channels are activated by intracellular Ca^2+^, it usually took several minutes for the intracellular environment to reach equilibrium after whole-cell configuration was established. The change in *I*_K_ amplitude was monitored using repetitive ramp pulses that were applied every 10 seconds (holding membrane potential −80 mV; test potentials from +20 mV to −120 mV for 400 ms). Once *I*_K_ became steady, a step-pulse protocol was also used to record baseline *I*_K_ (holding membrane potential −80 mV; test potentials in 10 mV steps from −140 mV to +40 mV for 300 ms). While *I*_K_ were monitored with ramp-pulses, amiodarone in various concentrations was applied to the bath solution. After maximum inhibition of *I*_K_ was achieved, above step pulse protocol was applied again to record the *I*_K_ under drug effect. After recordings with amiodarone were obtained, the drug was washed out. Finally, apamin (100 nM) was applied to ensure the measured *I*_K_ were apamin-sensitive. *I*_K_ after 100 nM apamin application were subtracted from the *I*_K_ under no drug application and the current difference was defined as *I*_KAS_ that is carried by SK2 channels. Inhibition of *I_K_* under various concentrations of amiodarone was compared and normalized to the inhibition of *I_K_* with 100 nM apamin (i.e., *I*_KAS_).

### Drugs and Reagents

Apamin was purchased from Tocris (catalog#1652), and was dissolved in water for a 500 µM stock solution. Amiodarone-HCl was also purchased from Tocris (catalog# 4095), and was dissolved in DMSO or ethanol for a 25 mM stock solution. All other chemicals were purchased from Sigma (St. Louis, MO).

### Ethics Statement

The protocol for animal experiments was approved by the Indiana University Institutional Animal Care and Use Committee.

### Data Analysis

Patch-clamp data were analyzed using Clampfit (Molecular Devices/Axon, Sunnyvale, CA). Inhibition of *I*_K_ with amiodarone was compared and normalized to *I*_KAS_ (*I*_K_ with 100 nM apamin) and was plotted as a function of amiodarone concentration. The data were fitted with the Hill equation: y = 1/[1+(IC_50_/x)^n^], where y indicates normalized *I*_KAS_, x is concentration of amiodarone, IC_50_ is concentration of amiodarone at half-maximal inhibition of *I*_KAS_ and n is the Hill coefficient. Curve fitting was performed by the method of least squares with data points weighted by the inverse of their variance with Igor Pro 6 (WaveMetrics, Lake Oswego, OR) and Prism 5 (GraphPad Software, La Jolla, CA).

### Statistical Analysis

Comparison of continuous variables between two groups was performed using Mann-Whitney-Wilcoxon test. Continuous variables among three groups were compared with Kruskal-Wallis test with post-hoc Dunn's multiple comparison test to compare differences between any two groups. All comparisons were performed using two-tailed tests and a *p* value less than 0.05 was considered statistically significant. Statistical analyses were performed using SPSS PASW Statistics 17 software (IBM, Chicago, IL) and Prism 5 (GraphPad Software, La Jolla, CA). Data in text and figures are presented as median [25th percentile; 75th percentile] or mean ± S.D. unless otherwise stated.

## Results

### Amiodarone inhibits *I*_KAS_

**Figure 1:**
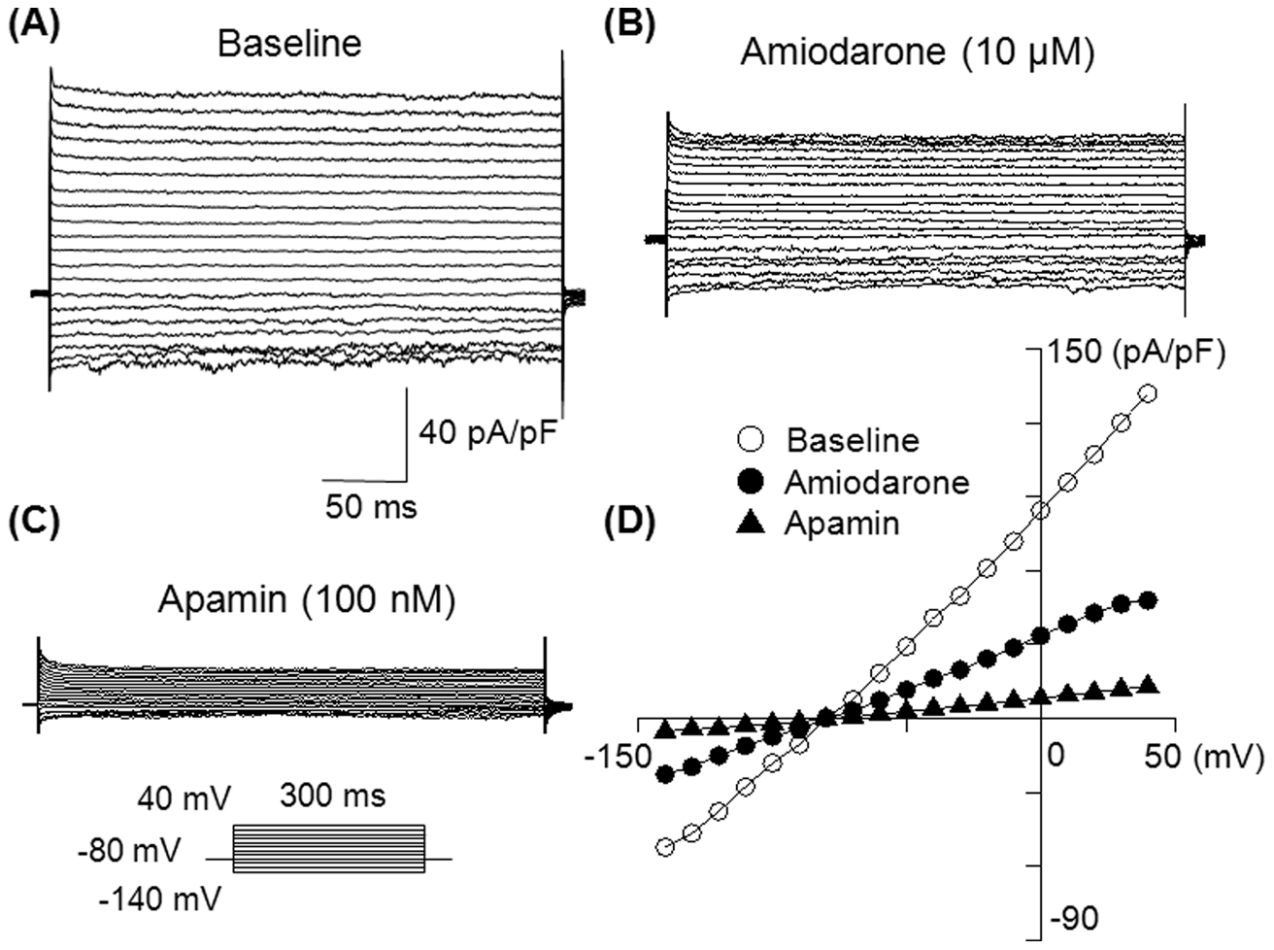
Inhibitory effect of amiodarone on *I*_KAS_. (A) Representative superimposed whole-cell *I*_K_ traces obtained by the pulse protocol shown in the inset. The pipette solution contained 1 µM free Ca^2+^ to activate *I*_KAS_. (B) Superimposed *I_K_* traces in the presence of 10 µM amiodarone. (C) Superimposed *I*_K_ traces after adding 100 nM apamin to the same preparation. (D) Current-voltage (*I-V*) relationships obtained in the absence and presence of 10 µM amiodarone, then 100 nM apamin. *I*_K_ was measured between 280 ms and 290 ms of the test pulse, and plotted against membrane potentials. Similar results with 10 µM amiodarone were observed in 5 cells.

We first tested whether the extracellularly applied amiodarone can inhibit *I*_KAS_. *I*_K_ was activated with various intrapipette Ca^2+^ concentrations. **Figure 1** shows representative traces of 1 µM Ca^2+^-activated *I_K_* obtained with a step-pulse protocol in the absence (**Figure 1A**) and in the presence of 10 µM amiodarone (**Figure 1B**) and 100 nM apamin (**Figure 1C**). Amiodarone significantly decreased *I*_K_ in a time-independent manner. **Figure 1D** illustrates the current-voltage (I-V) relationship in the absence and presence of these drugs. Inhibition by amiodarone (10 µM) was observed at all membrane potentials.

### Inhibition of *I*_KAS_ with amiodarone is reversible

**Figure 2:**
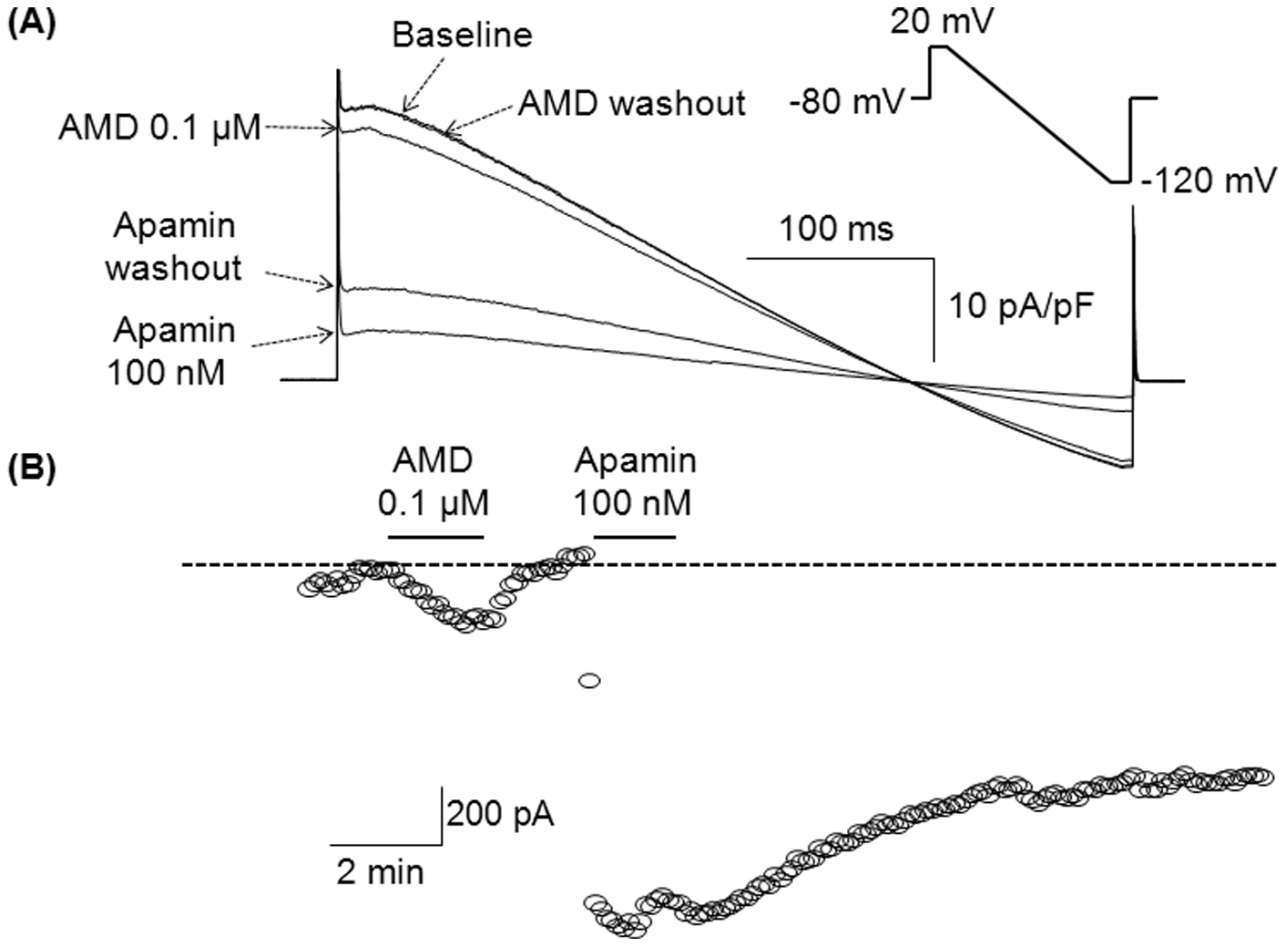
Inhibition of *I*_KAS_ with amiodarone is reversible. (A) Superimposed *I*_K_ traces in conditions as labeled. *I*_K_ was obtained with the ramp pulse protocol shown in the inset, and normalized with cell-capacitance. Intrapipette Ca^2+^ was 1 µM. (B) Time course of *I*_K_ measured at a membrane potential of 20 mV. The dotted line indicates the level of baseline *I*_K_. AMD indicates amiodarone.

We next tested whether the inhibitory effect of amiodarone was reversible. *I*_K_ was recorded with a repetitive ramp pulse protocol. **Figure 2A** shows representative *I*_K_ traces obtained in the absence (baseline) and in the presence of amiodarone (0.1 µM) and apamin (100 nM). While the inhibitory effect of amiodarone was completely reversed after washout, the inhibitory effect of apamin was only partially reversible. **Figure 2B** demonstrates the time course of *I_K_* measured at a membrane potential of 20 mV. We also performed experiments in which apamin (100 nM) was added first, followed by amiodarone administration. In these experiments, amiodarone did not further reduce the total *I*_K_.

### Inhibition of *I*_KAS_ with amiodarone is dose-dependent

**Figure 3:**
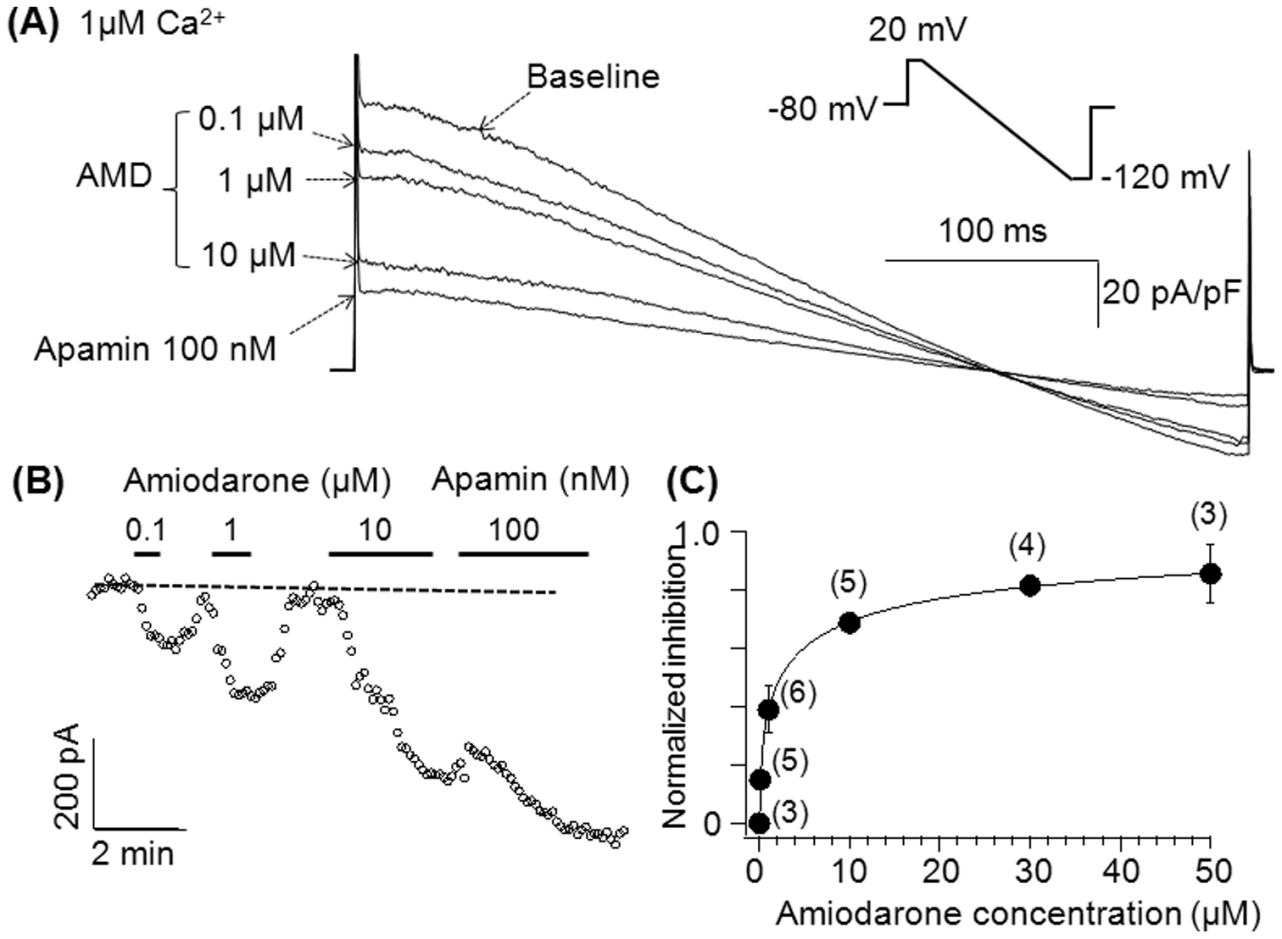
Inhibition of *I*_KAS_ with amiodarone is dose-dependent. (A) *I*_KAS_ was induced by the ramp pulse protocol used in Figure 2. (B) Time-course of *I*_K_ measured at 0 mV in the absence and presence of various amiodarone concentrations and apamin. The dotted line indicates the level of baseline *I*_K_. (C) Dose-dependent inhibitory effects of amiodarone on *I*_KAS_. The numbers in parenthesis indicate number of patches. The error bars represent S.E.

To study whether amiodarone's effects on *I*_KAS_ were dose-dependent, various consecutive concentrations of amiodarone were applied to the chamber after achieving steady-state *I*_KAS_ with repetitive ramp-pulse protocols. *I*_KAS_ was induced with an intrapipette Ca^2+^ concentration of 1 µM. As indicated in **Figure 3A**, *I*_KAS_ was inhibited by amiodarone in a dose-dependent manner. In **Figure 3B**, *I*_KAS_ is shown in the presence of various concentrations of amiodarone obtained at a membrane potential of 20 mV and is plotted as a function of time. **Figure 3C** shows the dose-dependency of the inhibition of *I*_KAS_ by amiodarone. The inhibition of *I*_KAS_ with various amiodarone concentrations was normalized to the inhibition with 100 nM apamin (i.e. total *I*_KAS_), and plotted as a function of amiodarone concentration. Data were fit with the Hill equation, yielding an IC_50_ of 2.67±0.25 µM, and a Hill coefficient of 0.51±0.02.

### Inhibition of *I*_KAS_ with amiodarone is affected by intracellular Ca^2+^ concentration

**Figure 4:**
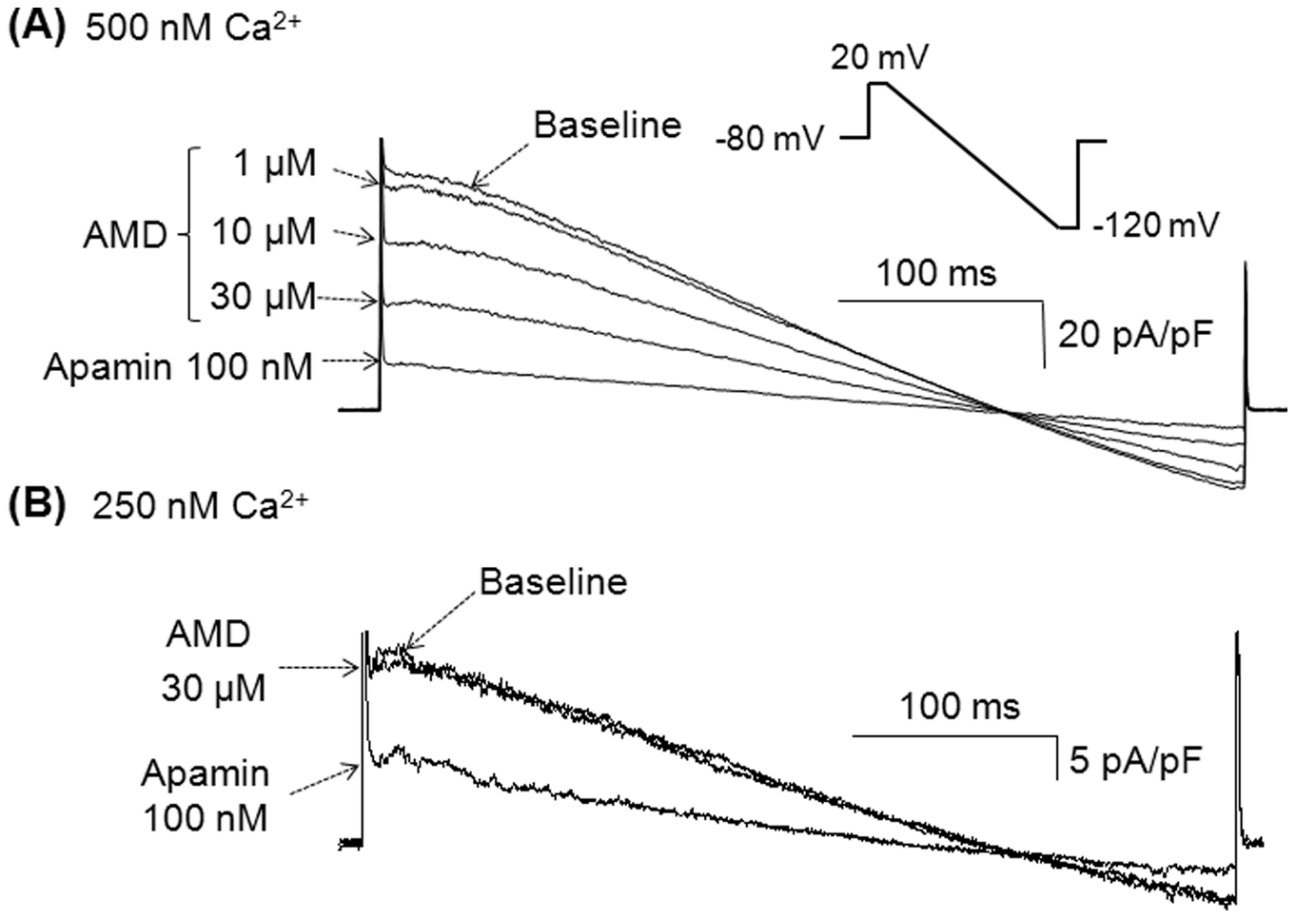
Inhibition of *I*_KAS_ with amiodarone is state-dependent. (A) Superimposed *I*_K_ traces in various conditions as labeled. The intrapipette Ca^2+^ was 500 nM. *I*_K_ was obtained with the same ramp pulse protocol used in Figure 2 (also shown in the inset). (B) Superimposed *I*_K_ traces induced with 250 nM intrapipette Ca^2+^. Similar results were observed in 5 cells. AMD indicates amiodarone.

The open-probability of SK2 channels is dependent on intracellular concentration of Ca^2+^ which is suggestive of different conformational states for SK2 channels at different Ca^2+^ concentrations [19]. Therefore, we questioned whether the extent of inhibition might also be Ca^2+^-dependent. To test this hypothesis, we induced *I*_KAS_ with various intrapipette Ca^2+^ concentrations and studied its inhibition by amiodarone. **Figure 4A** shows representative *I*_K_ traces induced with an intrapipette Ca^2+^ concentration of 500 nM at baseline and in the presence of various concentrations of amiodarone. Amiodarone exerts less *I*_KAS_ inhibition, when *I*_KAS_ is induced with 500 nM intrapipette Ca^2+^ compared to its inhibition of the *I*_KAS_ induced with 1 µM intrapipette Ca^2+^ (**Figure 3A**). Furthermore, the inhibitory effects of amiodarone (30 µM) on *I*_KAS_ were even smaller when the currents were induced with 250 nM rather than with 500 nM intrapipette Ca^2+^ (**Figure 4B**).

**Figure 5:**
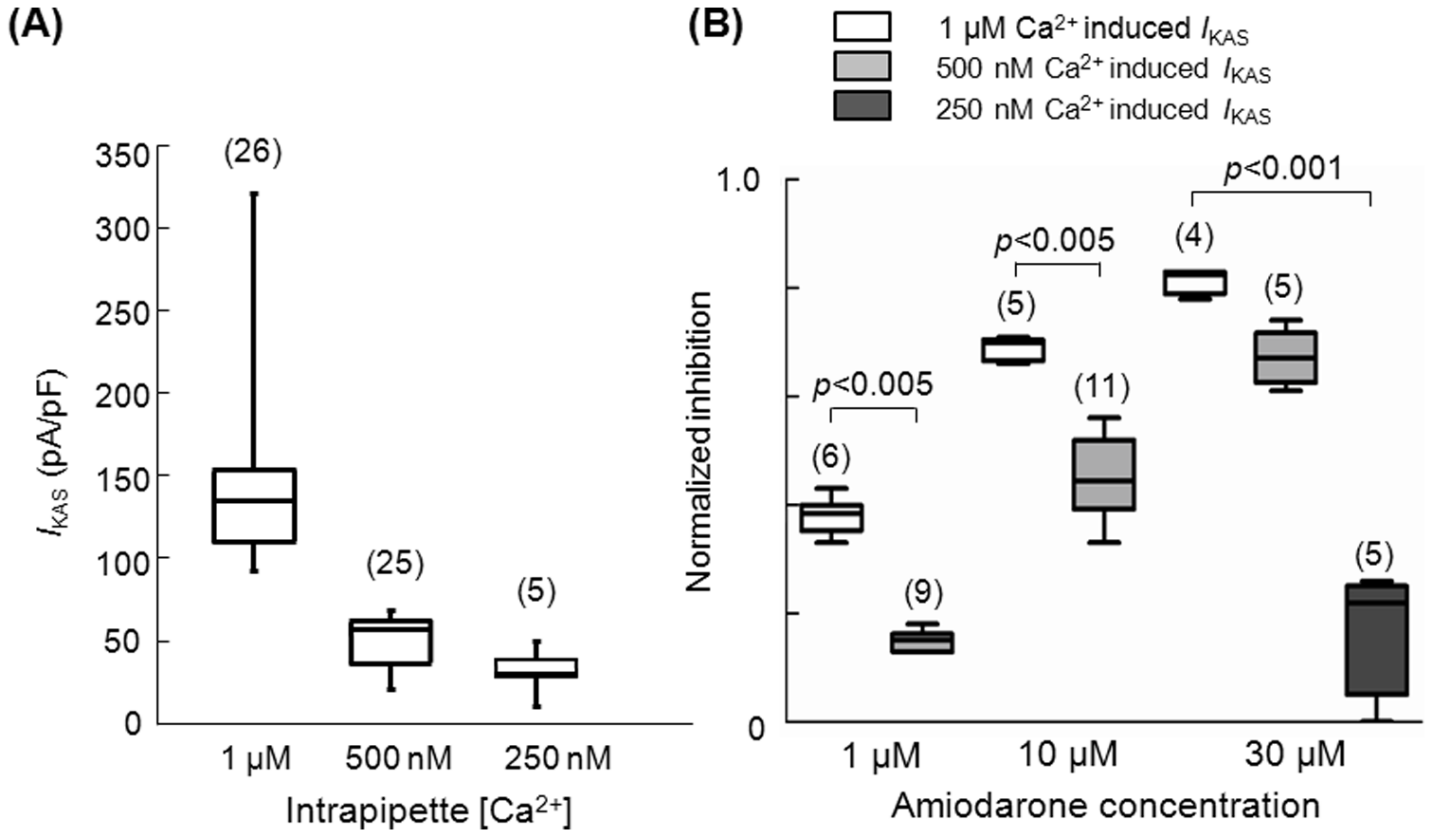
Density of *I*_KAS_ and its inhibition by amiodarone is dependent on the intracellular Ca^2+^ concentration. (A) *I*_KAS_ densities obtained with three different intrapipette Ca^2+^ concentrations. The box-plot indicates the median and 25 and 75 percentile values. The whiskers indicate the minimum and maximum values. (B) Inhibitory effects of various concentrations of amiodarone on *I*_KAS_ induced with various intrapipette Ca^2+^ concentrations as shown in the top labels. In the left panel, inhibitory effects of 1 μM amiodarone were tested on *I*_KAS_ induced with two different intrapipette Ca^2+^ concentrations (1 µM and 500 nM). In the mid panel, inhibitory effects of 10 μM amiodarone were tested on *I*_KAS_ induced with two different intrapipette Ca^2+^ concentrations (1 µM and 500 nM). In the right panel, inhibitory effects of 30 μM amiodarone were tested on *I*_KAS_ induced with three different Ca^2+^ concentrations (1 µM, 500 nM and 250 nM). Inhibition of 250 nM Ca^2+^ induced *I*_KAS_ by 1 µM and 10 µM amiodarone is not shown since this was almost undetectable. The numbers in parentheses indicate the number of patches.

**Figure 5A** shows *I*_KAS_ densities induced by various intrapipette Ca^2+^ concentrations. *I*_KAS_ density was significantly larger when induced with 1 µM Ca^2+^ compared to 500 nM and 250 nM. **Figure 5B** shows that the extent of amiodarone's inhibitory effect on *I*_KAS_ is dependent on whether the *I*_KAS_ is induced with 1 µM, 500 nM or 250 nM intrapipette Ca^2+^ (i.e. different channel conformations respond differently to amiodarone). We used three different therapeutic amiodarone concentrations (1, 10 and 30 µM) to inhibit *I*_KAS_. The extent of inhibition in each condition was normalized to the extent of inhibition achieved by 100 nM apamin of the1 µM intracellular Ca^2+^ induced *I*_KAS_. With both 1 μM and 10 μM amiodarone, the extent of inhibition we observed was significantly smaller when the currents were activated with 500 nM intrapipette Ca^2+^ (light grey boxes) compared to1 μM intrapipette Ca^2+^ (white boxes). The inhibition of *I*_KAS_ with 30 μM amiodarone was also highest when *I*_KAS_ was induced with 1 μM intrapipette Ca^2+^. Amiodarone, at 30 μM, inhibited the *I*_KAS_ induced by 500 nM Ca^2+^ less than by 1 μM Ca^2+^, and barely inhibited the *I*_KAS_ induced by 250 nM intrapipette Ca^2+^ (dark grey box on the far right). These results indicate that the inhibition of *I*_KAS_ by amiodarone is Ca^2+^ dependent.

### Desethylamiodarone, an amiodarone metabolite, also inhibits *I*_KAS_

**Figure 6:**
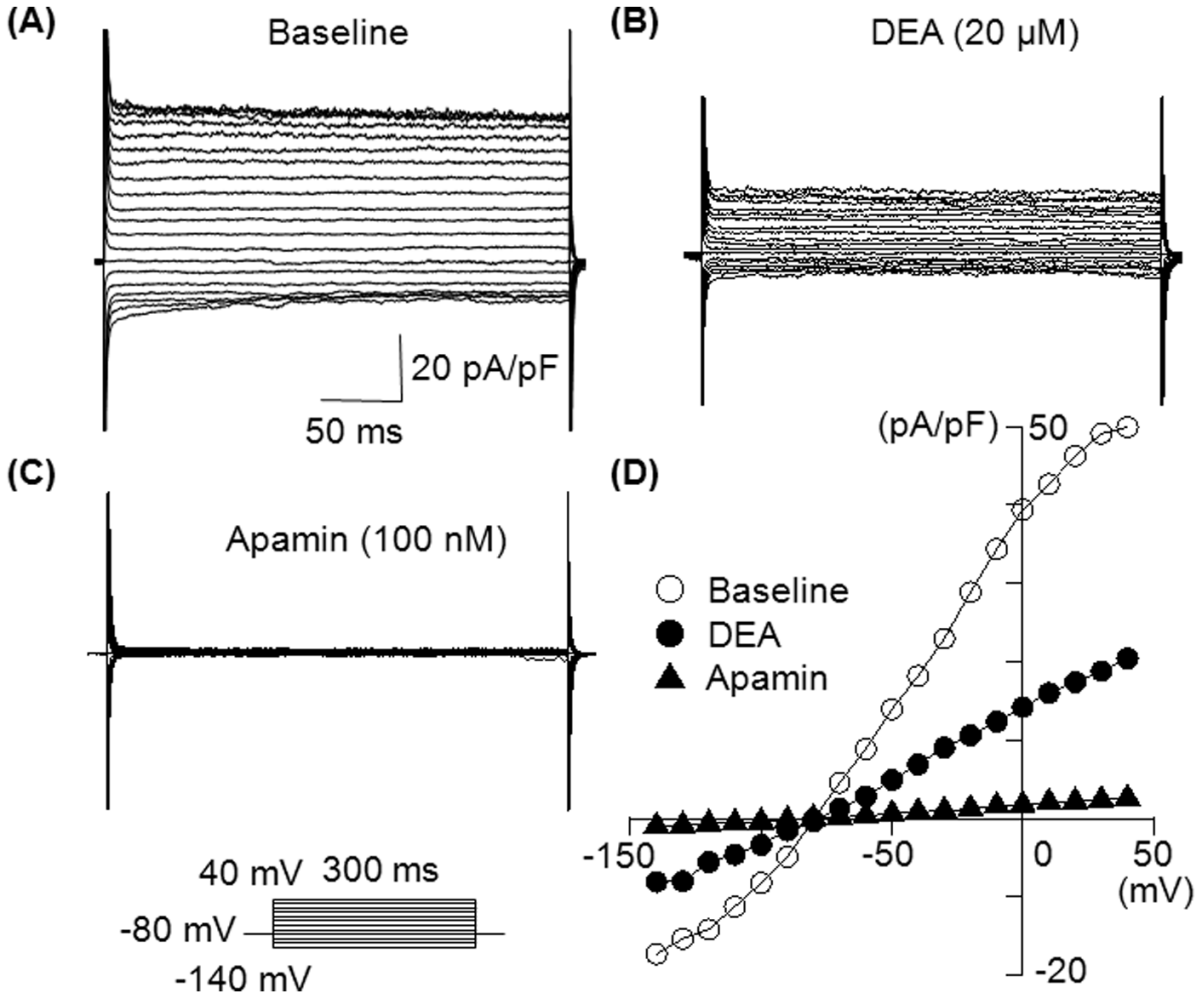
Desethylamiodarone also inhibits *I*_KAS_. (A) Superimposed whole-cell *I*_K_ traces obtained by the same pulse protocol used in Figure 1. The intrapipette Ca^2+^ was 1 µM. (B) Current-voltage (I-V) relationships obtained in the absence and presence of 20 µM desethylamiodarone (DEA), and then 100 nM apamin. Similar results were observed in 6 cells.

Once amiodarone is absorbed in the human body, it is extensively metabolized in the liver by cytochrome P450 3A4. The main metabolite is desethylamiodarone (DEA), which also has antiarrhythmic properties as the parent compound [20]. Therefore, we also studied the effects of DEA on *I*_KAS_. **Figure 6A-C** shows representative *I*_K_ traces obtained with a step-pulse protocol in the absence of DEA (**Panel** **A**), in the presence of 20 µM DEA (**Panel B**) and after addition of 100 nM apamin (**Panel C**). **Figure 6D** illustrates the current-voltage (I-V) relationships of these currents. Similar to amiodarone, DEA inhibited *I*_KAS_ in a time and voltage independent manner. The extent of inhibition with DEA (20 µM) was 79.0% [74.3; 86.8] of 1 µM intrapipette Ca^2+^ induced *I*_KAS_ (n = 6).

## Discussion

In this study, we examined the modulatory effects of amiodarone on human SK2 channels. Our novel findings are: (1) amiodarone reversibly inhibits the SK2 channels in a dose-dependent manner; (2) the inhibitory effect of amiodarone is time-independent and voltage independent; (3) the degree of I_KAS_ inhibition by amiodarone is dependent on the intracellular calcium concentration; and (4) desethylamiodarone, the main metabolite of amiodarone, also inhibits SK2 channels.

### Comparison with previous studies

Since the role of SK2 channels in ventricular arrhythmias of the failing heart had not been fully elucidated, effects of antiarrhythmic agents on SK2 channels were not studied systematically. Most studies about SK2 channels come from the neuroscience literature. Dreixler *et al.* [21] studied the effects of anesthetic agents on these channels and found that lidocaine inhibits SK2 channels expressed in HEK-293 cells. However, the IC_50_ they reported was 4.44 mM, which is much higher than the antiarrhythmic therapeutic concentration range (in µM) [22]. Although the underlying molecular mechanisms of lidocaine's effect on SK2 channels were not elucidated in the study, it was speculated that lidocaine interfered with the regulation of intracellular Ca^2+^, resulting in a change in SK2 activation. In our study, the intracellular Ca^2+^ concentration was clamped through the pipette solution and the extracellular buffer did not contain Ca^2+^. Therefore, it is unlikely that amiodarone inhibited SK2 channels by affecting intracellular Ca^2+^ in HEK-293 cells.

Very recently, Diness *et al.* [23] showed that three SK channel inhibitors (UCL1684, *N*-(pyridin-2-yl)-4-(pyridin-2-yl) thiazol-2-amine (ICA), and NS8593) successfully prevented the initiation of AF in various animal models. Since amiodarone (10 µM) also effectively prevented AF initiation in their models, they tested effects of 10 µM amiodarone on whole-cell SK2 currents expressed in HEK-293 cells. Surprisingly in their study, amiodarone did not show any significant effect on SK2 currents. Although detailed experimental conditions were not described in their paper, their previous study [24] indicated that the authors used 400 nM intrapipette Ca^2+^ to induce SK2 currents. Our study demonstrated that the inhibition of *I*_KAS_ with 30 µM amiodarone was significantly smaller when *I*_KAS_ was induced with 250 nM intracellular Ca^2+^ compared to 500 nM and 1 µM Ca^2+^, and inhibition with 10 µM amiodarone was also significantly smaller when *I*_KAS_ was induced with 500 nM Ca^2+^ compared to 1 µM Ca^2+^_._ Since open probability and state of SK2 channels are drastically different at various intracellular calcium concentrations below 1 µM [25], amiodarone's inhibitory effects may also be different. In other words, 400 nM intrapipette Ca^2+^ may not induce sufficient SK2 currents to test the inhibitory effects of amiodarone at 10 µM concentration. In addition, it appears that Diness *et al.* may have used nonphysiological K^+^ concentrations as their study showed a reversal potential of 0 mV. If the authors had used physiological K^+^ concentrations (e.g., [K^+^]_o_/ ([K^+^]_i_  = 4/154 mM) in whole-cell experiments as they referenced [24], the outward SK2 currents would have been induced at the holding potential of 0 mV and the reversal potential would have been around −90 mV.

### Antiarrhythmic effects of amiodarone

Since amiodarone affects various ion currents (e.g., *I*_Na_, *I*_Ca,L_, *I*_Ks_, *I*_Kr_, and *I*_to_) as well as β-adrenergic receptors, it is not straightforward to determine its most clinically important target for its anti-arrhythmic activity. Our study adds a new target ion channel to this list.

One mechanism for amiodarone's effectiveness in the treatment of electrical storm can be through prevention of excessive APD shortening after successful defibrillation. Persistence of Ca^2+^ elevation after the conclusion of repolarization results in depolarizing Na^+^ currents through the Na^+^/Ca^2+^ exchanger, which in turn results in late phase 3 EADs and triggered activity [13], [26]–[28]. Targeting the mechanism behind post-shock APD shortening would act as a specific therapeutic maneuver to prevent ES. In a recent study, we were able to effectively prevent spontaneous VF after defibrillation by the selective SK channel inhibitor apamin [13]. Using the same rabbit heart failure model, we demonstrated that amiodarone can decrease post-shock APD shortening, which underlie its effectiveness in treatment of ES (**Figure S2 in Data S2**).

Another mechanism behind amiodarone's effectiveness for ES could be through its effects on APD restitution. Previously, Omichi *et al*. reported that amiodarone flattened the APD restitution slope which in turn resulted in termination of VF in isolated swine ventricles [29]. Since VF induces Ca^2+^ accumulation due to high frequency depolarizations [30]–[33], it is reasonable to speculate that amiodarone's inhibitory action on SK2 channels are responsible for flattening of the APD restitution slope. This hypothesis is supported by a recent study that showed apamin flattens APD restitution curve at fast pacing rates [34].

We found that the inhibitory effects of amiodarone on SK2 channels were state-dependent (i.e., more inhibition for more current). Cytoplasmic domains of SK2 channels bind to calmodulin, and binding of Ca^2+^ to calmodulin may affect the conformation of SK2 channels and its gating kinetics [35]. This Ca^2+^-dependent conformational change may contribute to the state-dependent inhibition of SK2 by amiodarone. The state-dependent inhibition may be clinically important, since amiodarone may affect the channel only when the intracellular Ca^2+^ is elevated. This makes it particularly important in patients with heart failure or electrical storm where the basal intracellular Ca^2+^ is elevated.

In our study, an IC_50_ of amiodarone on whole-cell *I*_KAS_ induced with 1 µM Ca^2+^ was 2.67 µM. Since acute and chronic amiodarone administration results in plasma levels of approximately 0.16 to 10 µM [36], inhibitory effects of amiodarone on SK2 channels can be seen with doses used in the clinical setting. Le Bouter *et al*. [37] demonstrated that mice fed with clinically used doses of amiodarone for 6 weeks showed significant increase of SK2 mRNA levels in their total heart tissue. Although *I*_KAS_ were not determined in that study, it is reasonable to hypothesize that the transcripts could have increased as a compensatory mechanism to the inhibition of SK2 channels with chronic amiodarone therapy.

### Study Limitations

Our study has several limitations: (1) we studied only one variant of SK2 channel (*KCNN2* transcript variant 1). Several different SK2 variants are known, although cardiac expression of these variants remains unclear. In addition, other subtypes of SK channels (e.g., SK1 and SK3) exist in cardiomyocytes, and they may form heteromers with SK2 channel resulting in different sensitivity to amiodarone [38] in human hearts; (2) Clearly cytoplasmic environment of HEK-293 cells are very different from human cardiomyocytes. Since SK2 channels are regulated by various signal transduction pathways such as calmodulin, protein kinase CK2, and protein phosphatase 2A [35], effects of amiodarone on SK2 currents may differ between HEK-293 cells and human cardiomyocytes. To overcome this problem, testing amiodarone's effects on SK2 currents in cardiomyocytes or whole heart would have been helpful. However, since amiodarone inhibits various ion channels including *I*_Kr_, *I*_Ks_ and *I*_to_ in cardiomyocytes, specific inhibitors of these channels need to be used to isolate amiodarone's effects on SK2 currents. After attempting to study amiodarone effects in cardiomyocytes, we found that specific inhibitors of delayed rectifier K^+^ channels (*I_K_*_s_ and *I_K_*_r_), chromanol 293B and E4031 [39], [40] also inhibit SK2 currents complicating the interpretation of data we would obtain using cardiomyocytes (**Figure S3 in Data S3, Figure S4 in Data S4**). To avoid the non-specific effects of various “specific” ion channel inhibitors, genetically manipulated models are needed. However, it is beyond a scope of this study; (3) Currently we do not know whether apamin can prevent electrical storm in human since it is a neurotoxin and cannot be used in the clinical setting. However, our recent study using rabbit heart failure models demonstrated that *I*_KAS_ were upregulated and resulted in shortening of the APD after VF termination, predisposing the failing heart to spontaneous recurrence of VF. Apamin, through inhibition of SK2 channels, prevented the APD shortening, recurrence of VF and electrical storm. Similarly, we have shown that SK2 currents were upregulated in failing human ventricular myocytes compared with the non-failing myocytes, and Apamin prolonged the APD in failing human myocytes but not in the non-failing ones [12]. These facts, taken together, allow us to speculate that the inhibition of SK2 currents with amiodarone may, at least in part, underlie amiodarone's effects in termination and prevention of ES in human; (4) We assumed that the SK2 expression level is similar among different experiments, and thus *I*_KAS_ density can reflect the open probability of the channel. Our assumption was based on our consistent cell culture and transfection protocols. However, we do not have single-channel recording data. Therefore, we do not know the detailed molecular mechanisms behind this inhibition. In spite of these limitations, the results of this study showed for the first time that amiodarone is a potent inhibitor of *I*_KAS_. It is possible that *I*_KAS_ inhibition may partially account for the antiarrhythmic efficacy of amiodarone.

## Supporting Information

Data S1 **Control experiments. Figure S1.** Superimposed *I*_K_ traces obtained from an HEK-293 cell with mock transfection using the pEGFP-C3 vector. The *I*_K_ were recorded with an intra-pipette Ca^2+^ concentration of 1 µM. The voltage was clamped by the ramp-pulse protocol shown in the inset. Eighty repetitive ramp-pulses were applied every 10 seconds for approximately 5 minutes after the formation of whole-cell configuration.
(TIF)

Data S2 **Effects of amiodarone on the post-shock action potential durations.** We sought to investigate whether amiodaorone can antagonize the post-shock action potential duration (APD) shortening. For this, we used pacing-induced rabbit heart failure models since induction of ventricular fibrillation (VF) was difficult in human ventricle wedge preparations. The protocol was approved by the Indiana University Institutional Animal Care and Use Committee. Pacing-induced rabbit heart failure model was created and optical mapping studies in the Langendorff-perfused hearts were performed as previously described [1]. Failing hearts were stained with RH237 for measurement of the membrane potential (*V*_m_). Rapid ventricular pacing and 3 to 5 ventricular fibrillation-defibrillation episodes were mapped in each heart. Amiodarone (10 μM) was added to the perfusate for 30 minutes and then the same protocol was repeated. **Figure S2A** shows a representative action potential (AP) recording at a pacing cycle length (PCL) of 300 ms obtained from the anterobasal left ventricle without amiodarone. The average APD_80_ obtained was 176 ms. VF was induced by programmed electrical stimulation, and the fibrillating heart was defibrillated with DC current at 130–230 V. First post-shock APD was 141 ms with a preceding diastolic interval of 478 ms (**Figure S2B in Data S2**). Next, same protocol was repeated in the presence of amiodarone (10 µM). The average APD_80_ was 182 ms (**Figure S2C in Data** **S2**). **Figure S2D** shows a representative AP recording after defibrillation. First post-shock APD was 162 ms with a preceding diastolic interval of 496 ms. VF was induced several times, and post-shock APDs were measured. **Figure S2E** shows a plot of post-shock APDs as a function of preceding diastolic intervals. The data were analyzed with simple linear regression. The slope was not significantly affected by amiodarone (baseline: 0.04±0.01, n = 12 beats from two animals; amiodarone, 0.02±0.02, n = 13 beats, *p* = 0.158). On the contrary, y-intercept was longer in the presence of amiodarone compared to the baseline (baseline: 116.3±3.8, n = 12 beats; amiodarone: 145.3±5.0, n = 13 beats, *p*<0.0001). Percent shortening of APD at a PCL of 300 ms was 26.5% at baseline and 15.8% in the presence of amiodarone. **Figure S2. Amiodarone partially antagonizes post-shock APD shortening in failing hearts.** (A) Baseline AP recordings in the absence of amiodarone. (B) Post-shock AP recordings in the absence of amiodarone (10 µM). Three beats of VF was shown. DC depicts direct current shock. (C) Baseline AP recordings in the presence of amiodarone (10 µM). (D) Post-shock AP recordings in the presence of amiodarone. (E) Plot of post-shock APD_80_ as a function of preceding diastolic intervals.
(TIF)

Data S3 **Inhibitory effects of chromanol 293B on SK2 currents.** Amiodarone can inhibit various types of ion channels including slow and rapid components of the delayed rectifier K^+^ channels (*I*_Ks_ and *I*_Kr_), and the inward rectifier K^+^ channel I_K1_. In order to study the specific effects of amiodarone on SK2 currents as well as on the action potential (AP) in cardiomyocytes, the effects of amiodarone on other K^+^ channels need to be eliminated. To achieve this, we planned to use chromanol 293B and E-4031 to specifically block *I*_Ks_ and *I*_Kr_ [2]. However, the effects of these drugs on SK2 currents were unknown. Therefore, we studied the effects of 293B and E-4031 on SK2 currents expressed in HEK-293 cells. Unexpectedly, the most specific *I*_Ks_ blocker, chromanol 293B, blocked the *I*_KAS_ induced with 1 µM intra-pipette Ca^2+^ and repetitive ramp-pulse protocols (**Figure S3A in Data** **S3**). **Figure S3B** shows the dose dependent inhibition of *I*_KAS_ by chromanol 293B and its reversibility. Percent-inhibition of *I*_KAS_ with 100 µM chromanol 293B was 38.2±15.0% (n = 5). Since the IC_50_ of chromanol 293B on *I*_Ks_ is 10−30 µM, and 100 µM is necessary to fully block *I*_Ks_ [3], [4], we conclude that it is not feasible to use chromanol 293B to differentiate *I*_KAS_ from *I*_Ks_ in cardiomyocytes in order to study the effects of amiodarone on *I*_KAS_ and AP changes mediated by *I*_KAS_. **Figure S3.** **Effect of chromanol 293B on SK2 currents**. (**A**) Representative *I_K_* traces obtained with two different 293B concentrations. The ramp-pulse protocol used is shown in the inset. Note that apamin (100 nM) almost completely inhibited the *I*_K_ meaning that the *I*_K_ inhibited by 293B is *I*_KAS_. (**B**) The time course of the *I*_K_ measured at +20 mV. The *I_K_* reached steady state (baseline) within a few minutes of whole-cell configuration. Chromanol 293B inhibited the *I*_K_ in a dose-dependent manner. Subsequent application of apamin (100 nM) inhibited almost all of the *I*_K_.
(TIF)

Data S4 **Inhibitory effects of E4031 on SK2 currents.** Next, we tested the effect of E4031 (a specific *I*_Kr_ blocker) on *I*_KAS_. The IC_50_ of E4031 on tail *I*_Kr_ is 397 nM, and 3−5 µM of E4031 is necessary to block all *I*_Kr_ [2], [5]. Similar to 293B, E4031 also inhibited *I*_KAS_ induced with 1 µM intra-pipette Ca^2+^ and repetitive ramp-pulse protocols **(Figure S4A in Data** **S4). Figure S4B** demonstrates that the inhibition of *I*_KAS_ with E4031 is reversible. Percent-inhibition of *I*_KAS_ with 500 nM E4031 was 37.6±14.9% (n = 5). **Figure S4. Effect of E4031 on SK2 currents.** (**A**) Representative *I*_K_ traces in various conditions. The ramp-pulse protocol used is shown in the inset. (**B**) Time course of the *I*_K_ measured at +20 mV. The *I*_K_ reached steady state (dotted line) within a few minutes after the formation of whole-cell configuration. E4031 (0.5 µM) reversibly blocked the *I*_K_. Subsequent application of apamin (100 nM) inhibited most of the *I*_K_.
(TIF)

References S1 (DOCX)
