# Supplementary figures and images for: Amiodarone Inhibits Apamin-Sensitive Potassium Currents
Source: PLoS One. 2013 Jul 29;8(7):e70450. doi: 10.1371/journal.pone.0070450 (PMC3726612; doi:10.1371/journal.pone.0070450)

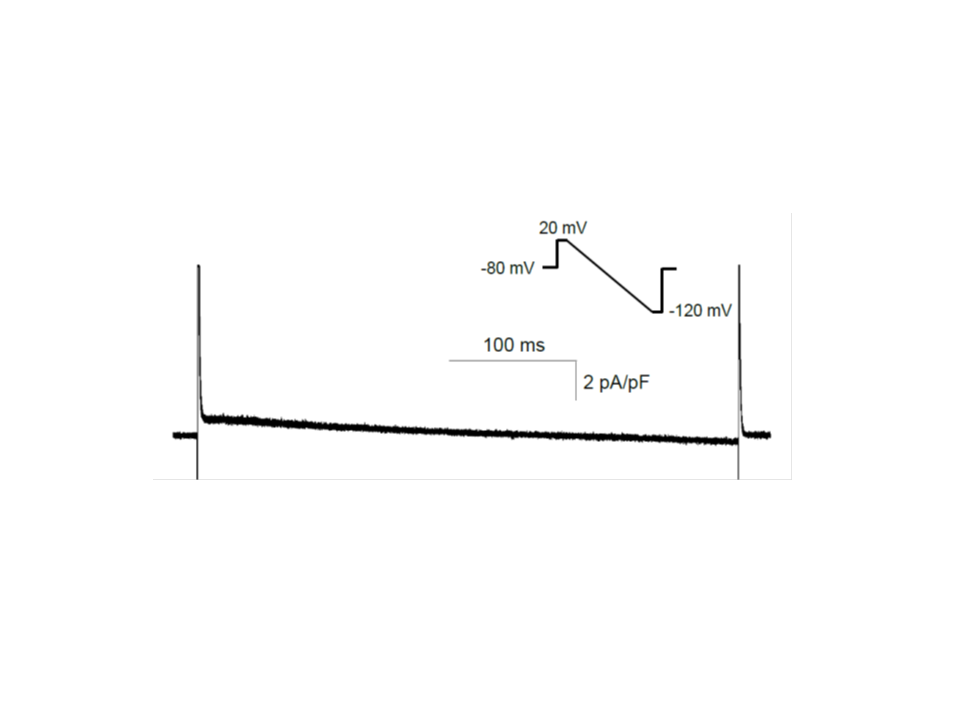

Supplement: Data S1 — Control experiments. Figure S1. Superimposed I K traces obtained from an HEK-293 cell with mock transfection using the pEGFP-C3 vector. The I K were recorded with an intra-pipette Ca2+ concentration of 1 µM. The voltage was clamped by the ramp-pulse protocol shown in the inset. Eighty repetitive ramp-pulses were applied every 10 seconds for approximately 5 minutes after the formation of whole-cell configuration. (TIF) [file pone.0070450.s001.tif]

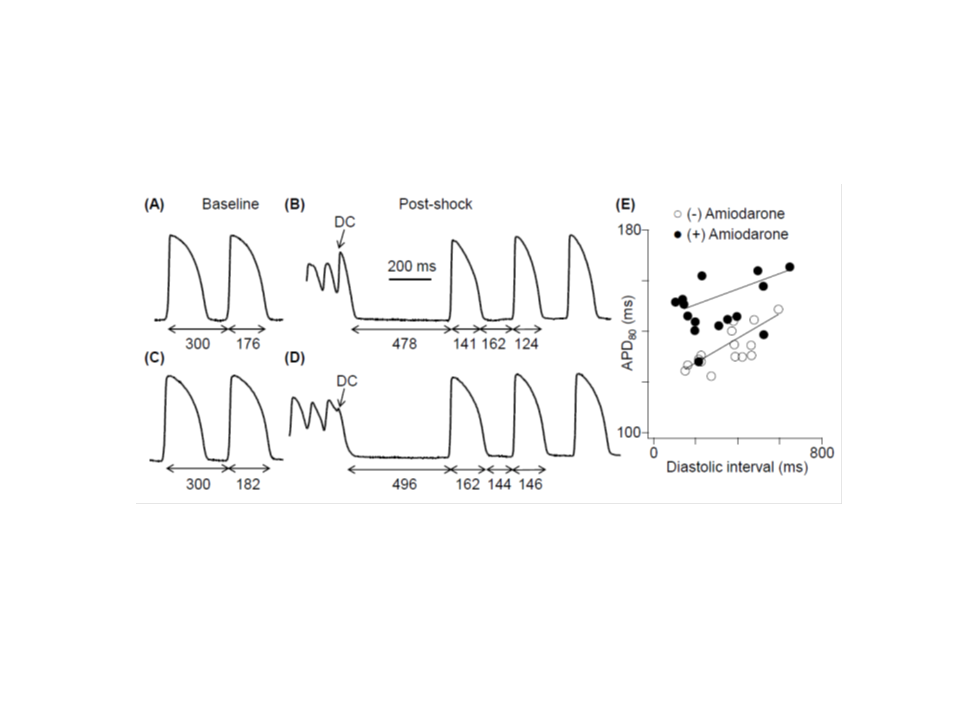

Supplement: Data S2 — Effects of amiodarone on the post-shock action potential durations. We sought to investigate whether amiodaorone can antagonize the post-shock action potential duration (APD) shortening. For this, we used pacing-induced rabbit heart failure models since induction of ventricular fibrillation (VF) was difficult in human ventricle wedge preparations. The protocol was approved by the Indiana University Institutional Animal Care and Use Committee. Pacing-induced rabbit heart failure model was created and optical mapping studies in the Langendorff-perfused hearts were performed as previously described [1]. Failing hearts were stained with RH237 for measurement of the membrane potential (V m). Rapid ventricular pacing and 3 to 5 ventricular fibrillation-defibrillation episodes were mapped in each heart. Amiodarone (10 μM) was added to the perfusate for 30 minutes and then the same protocol was repeated. Figure S2A shows a representative action potential (AP) recording at a pacing cycle length (PCL) of 300 ms obtained from the anterobasal left ventricle without amiodarone. The average APD80 obtained was 176 ms. VF was induced by programmed electrical stimulation, and the fibrillating heart was defibrillated with DC current at 130–230 V. First post-shock APD was 141 ms with a preceding diastolic interval of 478 ms (Figure S2B in Data S2). Next, same protocol was repeated in the presence of amiodarone (10 µM). The average APD80 was 182 ms (Figure S2C in Data S2). Figure S2D shows a representative AP recording after defibrillation. First post-shock APD was 162 ms with a preceding diastolic interval of 496 ms. VF was induced several times, and post-shock APDs were measured. Figure S2E shows a plot of post-shock APDs as a function of preceding diastolic intervals. The data were analyzed with simple linear regression. The slope was not significantly affected by amiodarone (baseline: 0.04±0.01, n = 12 beats from two animals; amiodarone, 0.02±0.02, n = 13 beats, p = 0.158). On the [file pone.0070450.s002.tif]

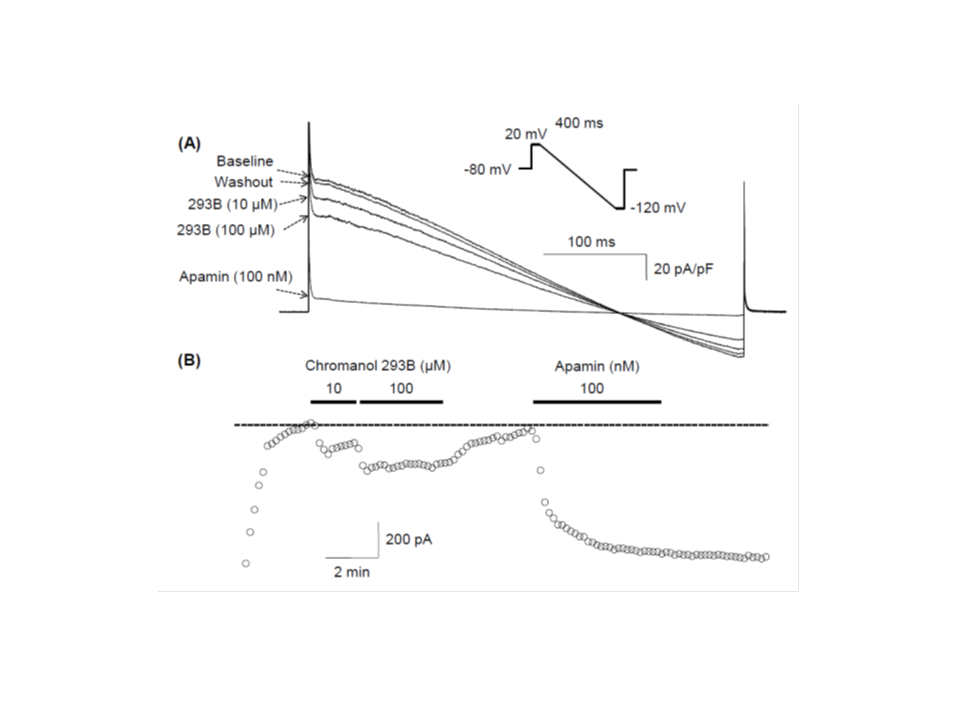

Supplement: Data S3 — Inhibitory effects of chromanol 293B on SK2 currents. Amiodarone can inhibit various types of ion channels including slow and rapid components of the delayed rectifier K+ channels (I Ks and I Kr), and the inward rectifier K+ channel IK1. In order to study the specific effects of amiodarone on SK2 currents as well as on the action potential (AP) in cardiomyocytes, the effects of amiodarone on other K+ channels need to be eliminated. To achieve this, we planned to use chromanol 293B and E-4031 to specifically block I Ks and I Kr [2]. However, the effects of these drugs on SK2 currents were unknown. Therefore, we studied the effects of 293B and E-4031 on SK2 currents expressed in HEK-293 cells. Unexpectedly, the most specific I Ks blocker, chromanol 293B, blocked the I KAS induced with 1 µM intra-pipette Ca2+ and repetitive ramp-pulse protocols (Figure S3A in Data S3). Figure S3B shows the dose dependent inhibition of I KAS by chromanol 293B and its reversibility. Percent-inhibition of I KAS with 100 µM chromanol 293B was 38.2±15.0% (n = 5). Since the IC50 of chromanol 293B on I Ks is 10−30 µM, and 100 µM is necessary to fully block I Ks [3], [4], we conclude that it is not feasible to use chromanol 293B to differentiate I KAS from I Ks in cardiomyocytes in order to study the effects of amiodarone on I KAS and AP changes mediated by I KAS. Figure S3. Effect of chromanol 293B on SK2 currents. (A) Representative IK traces obtained with two different 293B concentrations. The ramp-pulse protocol used is shown in the inset. Note that apamin (100 nM) almost completely inhibited the I K meaning that the I K inhibited by 293B is I KAS. (B) The time course of the I K measured at +20 mV. The IK reached steady state (baseline) within a few minutes of whole-cell configuration. Chromanol 293B inhibited the I K in a dose-dependent manner. Subsequent application of apamin (100 nM) inhibited almost all of the I K. (TIF) [file pone.0070450.s003.tif]

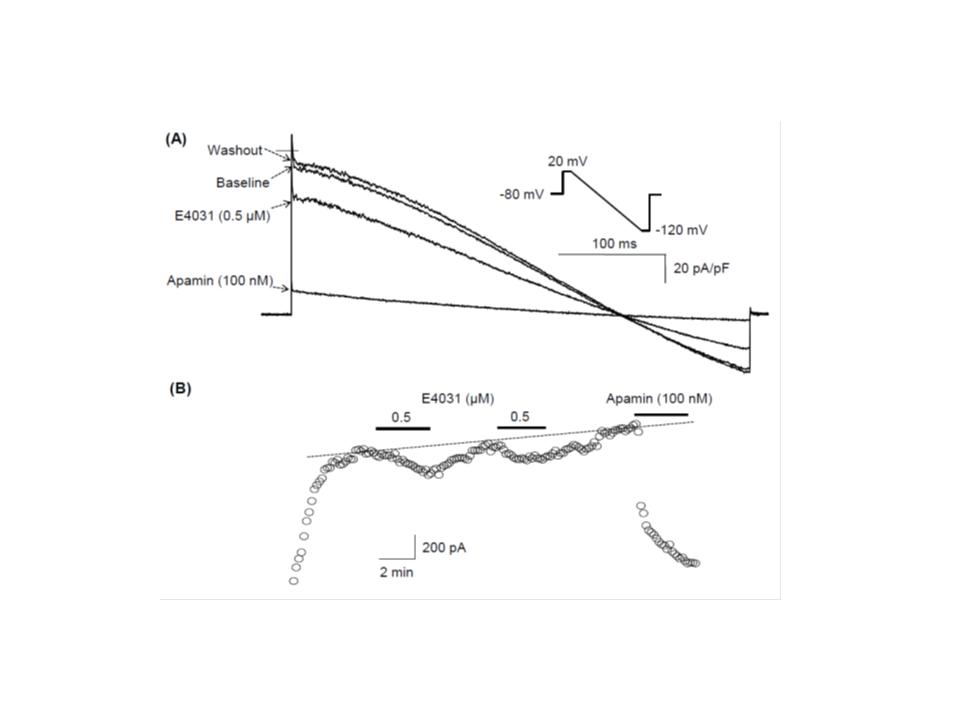

Supplement: Data S4 — Inhibitory effects of E4031 on SK2 currents. Next, we tested the effect of E4031 (a specific I Kr blocker) on I KAS. The IC50 of E4031 on tail I Kr is 397 nM, and 3−5 µM of E4031 is necessary to block all I Kr [2], [5]. Similar to 293B, E4031 also inhibited I KAS induced with 1 µM intra-pipette Ca2+ and repetitive ramp-pulse protocols (Figure S4A in Data S4). Figure S4B demonstrates that the inhibition of I KAS with E4031 is reversible. Percent-inhibition of I KAS with 500 nM E4031 was 37.6±14.9% (n = 5). Figure S4. Effect of E4031 on SK2 currents. (A) Representative I K traces in various conditions. The ramp-pulse protocol used is shown in the inset. (B) Time course of the I K measured at +20 mV. The I K reached steady state (dotted line) within a few minutes after the formation of whole-cell configuration. E4031 (0.5 µM) reversibly blocked the I K. Subsequent application of apamin (100 nM) inhibited most of the I K. (TIF) [file pone.0070450.s004.tif]
