# Supplementary material for: Amiodarone Inhibits Apamin-Sensitive Potassium Currents
Source: PLoS One. 2013 Jul 29;8(7):e70450. doi: 10.1371/journal.pone.0070450 (PMC3726612; doi:10.1371/journal.pone.0070450)
Supplement: References S1 — (DOCX) [file pone.0070450.s005.docx]

**Supporting Information**

**References**

1. Chua SK, Chang PC, Maruyama M, Turker I, Shinohara T, et al. (2011) Small-Conductance Calcium-Activated Potassium Channel and Recurrent Ventricular Fibrillation in Failing Rabbit Ventricles. Circ Res 108: 971-979.

2. Jurkiewicz NK, Sanguinetti MC (1993) Rate-dependent prolongation of cardiac action potentials by a methanesulfonanilide class III antiarrhythmic agent. Specific block of rapidly activating delayed rectifier K+ current by dofetilide. Circ Res 72: 75-83.

3. Seebohm G, Lerche C, Pusch M, Steinmeyer K, Bruggemann A, et al. (2001) A kinetic study on the stereospecific inhibition of KCNQ1 and I(Ks) by the chromanol 293B. Br J Pharmacol 134: 1647-1654.

4. Ding WG, Toyoda F, Matsuura H (2002) Blocking action of chromanol 293B on the slow component of delayed rectifier K(+) current in guinea-pig sino-atrial node cells. Br J Pharmacol 137: 253-262.

5. Sanguinetti MC, Jurkiewicz NK (1990) Two components of cardiac delayed rectifier K+ current. Differential sensitivity to block by class III antiarrhythmic agents. J Gen Physiol 96: 195-215.
